# Combined and sequential liver–kidney transplantation in children

**DOI:** 10.1007/s00467-017-3880-4

**Published:** 2018-01-10

**Authors:** Ryszard Grenda, Piotr Kaliciński

**Affiliations:** 1 Department of Nephrology, Kidney Transplantation & Hypertension, The Children’s Memorial Health Institute, Warsaw, Poland; 2 Department of Surgery & Organ Transplantation, The Children’s Memorial Health Institute, Warsaw, Poland

**Keywords:** Combined and sequential liver–kidney transplantation (CLKT and SLKT), Liver failure vs. enzymatic defect, Kidney after liver in transplant recipients

## Abstract

Combined and sequential liver–kidney transplantation (CLKT and SLKT) is a definitive treatment in children with end-stage organ failure. There are two major indications: - terminal insufficiency of both organs, or - need for transplanting new liver as a source of lacking enzyme or specific regulator of the immune system in a patient with renal failure. A third (uncommon) option is secondary end-stage renal failure in liver transplant recipients. These three clinical settings use distinct qualification algorithms. The most common indications include primary hyperoxaluria type 1 (PH1) and autosomal recessive polycystic kidney disease (ARPKD), followed by liver diseases associated with occasional kidney failure. Availability of anti-C5a antibody (eculizumab) has limited the validity of CLKT in genetic atypical hemolytic uremic syndrome (aHUS). The liver coming from the same donor as renal graft (in CLKT) is immunologically protective for the kidney and this provides long-term rejection-free follow-up. No such protection is observed in SLKT, when both organs come from different donors, except uncommon cases of living donation of both organs. Overall long-term outcome in CLKT in terms of graft survival is good and not different from isolated liver or kidney transplantation, however patient survival is inferior due to complexity of this procedure.

## Introduction

Combined liver–kidney transplantation (CLKT) is a procedure in which liver (whole or partial) and kidney allografts, coming from the same (deceased in most cases) donor, are transplanted to the pediatric recipient during a single surgical procedure. Sequential liver–kidney transplantation (SLKT) is divided into two distinct stages: isolated transplantation of the liver (or kidney) and (after a certain time interval) – transplantation of the kidney (or liver) allograft, coming from different (deceased) or the same living-related donors. This review describes aspects of surgical and post-transplant management, immunosuppression-related issues, several specific indications for CLKT and SLKT, as well as the outcomes of both procedures.

## Surgical aspects and immediate post-operative care

Combined liver and kidney transplantation (CLKT) is a very uncommon procedure (e.g., the overall number of annual number of transplantations worldwide in children with autosomal recessive polycystic kidney disease (ARPKD) usually does not exceed 30 cases), therefore should be performed by experienced surgical and multidisciplinary teams, as its complexity may be the reason of early failure with fatal consequences, especially in young children of low body weight [1]. CLKT is almost exclusively performed with organs from deceased donors. Although technically possible, procurement of part of the liver and a kidney from the same living donor is considered a risky procedure. It is more feasible when performed sequentially, usually the liver first and then the kidney [2]. In case of CLKT of organs procured from a deceased donor, the difference of body mass between donor and recipient should not exceed 50%. In children with portal hypertension and hypersplenism, removal of the large spleen may be necessary to make place for a relatively large liver graft. CKLT should not be accompanied by native nephrectomy in patients with ARPKD, as the extension of surgery increases the overall risk of procedural complications. Transplantation of the whole liver without its reduction avoids prolonged cold ischemia time for both liver and kidney, and reduces the risk of postoperative complications related to partial graft transplantation (like bleeding or bile leak). Keeping cold ischemia time as short as possible is crucial for immediate post-transplant function, thus reperfusion of both grafts should be done as early as possible. Cold ischemia time of the liver should not exceed 8–10 h and of the kidney graft should not be longer than 10–12 h. This is available with good center-driven logistics. The liver graft is transplanted first and after vascular anastomoses the liver is reperfused. Immediately after liver reperfusion, the kidney graft is implanted, anastomoses of the renal vein and artery in the “end to back” fashion to external or common iliac vessels are done, and the graft is reperfused. Single doses of mannitol (0.5 g/kg) and furosemide (3–5 mg/kg) are given at this moment to force the diuresis. The biliary and then ureterovesical anastomoses are done after reperfusion of both grafts and wounds are closed with the drains left around the transplanted organs.

In anuric patients, e.g., in children with ARPKD after bilateral native nephrectomy performed early in life [3], CLKT is more difficult in terms of intra-surgery fluid challenge. This is an indication to introduce continuous hemodialysis/hemodiafiltration (CVVHD/CVVHDF) in the operating theater and maintain this treatment during the whole surgery. Extracorporeal treatment is performed via single superior vena cava (SVC) double-lumen access or twin access with blood circulating from a catheter placed in the inferior vena cava (IVC) to a catheter introduced into the SVC. This helps to maintain blood pressure without fluid overload, particularly in the anhepatic phase, when the infrahepatic IVC is clamped. In cases of inadequate hourly diuresis, CVVHDF should be continued after transplantation, with no anticoagulation if possible. Citrate anticoagulation is safer compared to heparin-based anticoagulation in terms of the risk of bleeding, unless liver graft insufficiency increases the risk of citrate intoxication [4].

In cases of CLKT performed in patients with hyperoxaluria type 1 (PH1), the intensive hemodiafiltration should be continued after transplantation to remove the excess oxalate, intensively mobilized from the body deposits to plasma and urine. This approach is mandatory in cases of delayed renal graft function, however it might also be used in cases of immediate renal graft function in selected patients with severe systemic oxalosis and high oxalate burden. The aim is to protect the transplanted kidney against rapid recurrence of nephrocalcinosis. The optimal duration of post-transplant hemodiafiltration is not defined, however it is reasonable to monitor plasma oxalate concentration and maintain extracorporeal removal of oxalate until stable normalization below the saturation level (< 30 μmol/l) is achieved with good renal function [4–6].

During the whole CLKT surgery, particular attention should be given to achieving good surgical hemostasis and controlling coagulation. In patients with liver-related coagulopathy, the major disturbances should be corrected before surgery with fresh frozen plasma (FFP), cryoprecipitate, or platelets, if necessary. The infusion of tranexamic acid may be given to control fibrinolysis during surgery, particularly in patients with a history of previous abdominal surgeries or advanced portal hypertension with multiple intra-abdominal varices and hypersplenism, as in these cases native hepatectomy is difficult and associated with marked blood loss. One the other hand it is important not to overcorrect coagulation, as it may lead to vascular thrombotic complications after reperfusion of both organs, thus administration of FFP, platelets, cryoprecipitate, fibrinogen formulations, and other procoagulants during surgery should be limited to the minimum necessary. Apart from routine laboratory tests, the best intraoperative monitoring of coagulation during CLKT is provided by repeated thromboelastometry, which shows real clinical coagulation status and guides adequate treatment. In some cases the infusion of catecholamines (dopamine and norepinephrine) should be given to maintain adequate blood pressure without fluid overload.

Doppler ultrasound examination should be done in the operating room before wounds are closed to ensure adequate blood flow through vascular anastomoses. Early post-operation care requires staying in the pediatric intensive care unit as mechanical ventilation may be necessary for 1–2 days. Very close monitoring of the patient and both transplanted organs is mandatory in the early postoperative period. Very detailed fluid balance (both water-electrolyte and volume), including all drained losses and hourly urine output should be kept to promote perfusion of the grafts and maintain adequate diuresis from the transplanted kidney [4]. Central venous pressure (CVP) should be maintained within a range of 5–10 cm^.^H_2_O by supplementation of 100% of abdominal drainage volumes and hourly diuresis. Hematocrit is kept between 25 and 30% during the whole perioperative period and coagulation should not be corrected unless there is clinically symptomatic bleeding or the international normalized ratio (INR) exceeds 3–3.5. Administration of blood-driven products (packed red blood cells, fresh-frozen plasma, platelets, albumin) should be kept to a minimum and related to the current needs of the recipient, measured losses, and results of laboratory tests. Our center uses anticoagulants (fractionated or low molecular weight heparin) postoperatively for 7–14 days as routine antithrombotic prophylaxis.

Patency of vascular anastomoses should be checked twice daily with Doppler ultrasound during the first 7 days after transplantation and then less frequently until discharge.

In most clinical situations of sequential procedure (SLKT), the liver transplantation precedes kidney transplantation, with the exception of a few patients with renal polycystic disease and liver fibrosis. The transplantation surgery of each individual graft in SLKT is not different from combined transplantation and is just performed as two separate procedures. In SLKT, most often one of the organs may be procured from a living donor and another one from a deceased donor, however using the same living-related donor has also been reported [2].

## Immunoprotection and immunosuppression

### Immunoprotection

There are data suggesting a protective effect of the liver to the kidney when procured from the same donor and transplanted during one surgical procedure (CLKT), which translates to a low rate of acute rejection compared to isolated kidney or liver transplantation, and results in improved renal allograft survival [7, 8]. There are various suggested mechanisms behind observed clinical immunoprotection, related to ability of the liver to modify the recipient immune system. Circulating alloantibodies may be neutralized by soluble class I HLA antigens produced by the transplanted liver. The liver graft delivers soluble HLA-G antigen, exerting an inhibitory effect on natural killer (NK) and cytotoxic T cells. Kupfer cells of the liver graft are able to clear circulating anti-HLA antibodies. They may also be bound to the HLA glycoproteins presented on hepatic endothelial cells [9–11].

It should be noted that a study analyzing 2484 adult recipients of CLKT showed that patient (*P* = 0.002) and overall kidney graft survival (*P* = 0.015) were significantly diminished among sensitized patients. Differences in patient survival translated to an estimated half-life of 10.3 years among nonsensitized recipients, versus 7.8 years among sensitized recipients. Therefore, presensitization may need to be considered in a risk stratification of CLKT [12]. The recent report from the ESPN/ERA-EDTA Registry on outcomes of CLKT vs. isolated kidney transplantation in children with autosomal recessive polycystic kidney disease (ARPKD) does not show a protective effect of liver transplantation on renal graft survival [1]. Among our patients with ARPKD who received CLKT, including three cases who had lost previously transplanted kidneys due to chronic rejection, there was no episode of acute rejection in renal grafts (*n* = 15) and a single, mild episode of rejection in a liver graft at 5-year follow-up. However, none of those three retransplanted patients (with kidney in CLKT) could be classified as highly sensitized [13].

The protective effect has not been observed in sequential liver and kidney transplantation (SLKT), however some studies have indicated the importance of HLA matching of the second graft with the previous one (kidney after liver) for improved outcome. The better the HLA match between liver and kidney, the lower the further incidence of acute rejection of the renal graft [8, 14]. The protective effect of liver to kidney, coming from the same living-related donor and transplanted sequentially (SLKT; kidney after liver) to a pediatric recipient against positive cross-match and presence of donor-specific antibodies, has also been reported [15]. This effect of a good match on long-term outcomes has confirmed in a specific study, in which the same living-related donors were used for primary liver and then secondary kidney transplantation (SLKT; *n* = 13), with 100% (death-censored) 10-year graft survival [2]. Overall, it seems that clinically significant protective effect of liver to kidney in CLKT has some limitations and is mainly seen in nonsensitized patients, while in SLKT, is present only in cases where both organs were sequentially procured from the same living-related donor, which is a rare situation.

### Immunosuppression

There are no universal recommendations with regard to the choice of optimal immunosuppression in CKLT and SLKT patients, and the decision is based on the experience of a particular center. There are a variety of protocols reported, including one calcineurin inhibitor (cyclosporine; CsA or tacrolimus; TAC); one antiproliferative agent (azathioprine; AZA or mycophenolate mofetil; MMF); mTOR inhibitor (sirolimus; SIR) and steroids (prednisolone; Pred) in different combinations: - triple therapy (CsA/TAC+ MMF/AZA + Pred); − double therapy (TAC + Pred; SIR + Pred) or steroid-free regimens (TAC+ MMF; TAC monotherapy). Use of induction was also variable and both blocking (anti-IL2R ab; basiliximab or daclizumab) and administration of depleting (polyclonal or monoclonal) antibodies (thymoglobuline/alemtuzumab) have been reported [13, 16–21].

## Indications

There are three major groups of indications for liver–kidney transplantation. The first group includes cases where there is not irreversible liver failure, but specific isolated functional (metabolic or immunological) defect of the native liver is an underlying mechanism of systemic disease and renal failure is one of the clinical manifestations. The native liver with a single specific defect otherwise maintains other functions, therefore the liver allograft is transplanted exclusively as the source of the lacking enzyme. The second group includes end-stage liver failure of various underlying causes, including ciliopathies (such as autosomal recessive polycystic kidney disease with liver fibrosis), accompanied by concomitant renal failure. CLKT or SLKT may be performed in both settings, however the selection of modality depends on current individual status of both organs, general condition of the recipient, and center experience. The third group includes secondary chronic renal failure in recipients of non-renal solid organ transplant in whom sequential kidney (after liver) transplantation is necessary (usually) several years thereafter.

Based on data from the Scientific Registry of Transplant Recipients, the most common liver-related indication for CLKT was primary hyperoxaluria type 1 (PH1; 37%), followed by congenital liver fibrosis (accompanied by renal failure) (18%), other non-PH1 metabolic diseases (7%), and polycystic liver disease (5%), as well as various other liver-related disorders (each about 1%). The most common primary renal disease in patients undergoing CLKT was primary hyperoxaluria (oxalate nephropathy) (36%), followed by polycystic kidney disease (25%) [22]. Indications for liver–kidney transplantation are summarized in Table 1.

**Table 1:** Indications for pediatric liver–kidney transplantation in regard to the types of transplantation procedure

Group of indications Specific indications	CLKT	SLKT
Irreversible liver failure:	Simultaneous liver and kidney failure	Failure of one organ precedes failure of the other one
Autosomal recessive polycystic kidney disease (ARPKD)		
α1-antitripsin deficiency	Extremely rare	Extremely rare
Primary sclerotic cholangitis Alagille syndrome Boichis syndrome		
Lack/low activity of specific enzyme/regulator of immune system:	Recommended in cases of late diagnosis and renal failure (eGFR < 15 ml/min/1.73 m^2^)	Suggested to consider in cases of early diagnosis and relatively good native renal function (eGFR 15–29 ml/min/1.73 m^2^)
Hyperoxaluria type 1 (PH 1) Methylmalonic acidemia (MMA) Type 1a glycogen storage disease (von Gierke disease)		
Atypical HUS (aHUS) *	*currently not recommended with availability of eculizumab	*Possible rare indication in case of poor efficacy of eculizumab-based prophylaxis
Liver transplantation and further failure of native kidneys	Not applicable	eGFR < 15 ml/min/1.73 m^2^

*CLKT* combined liver–kidney transplantation, *SLKT* sequential liver–kidney transplantation, *ARPKD* autosomal recessive polycystic kidney disease, *eGFR* estimated glomerular filtration rate, *PH1* primary hyperoxaluria type I, *MMA* methylmalonic acidemia, *aHUS* atypical hemolytic uremic syndrome 2

### Primary hyperoxaluria type 1 and 2 (PH1, PH2)

The underlying genetic defect of primary hyperoxaluria type 1 (PH1)—mutation of the *AXGT* gene encoding enzyme alanine-glyoxylate aminotransferase—leads to systemic accumulation of oxalate deposits in many tissues, except the liver, where the enzyme is localized. Oxalate forms crystals with calcium in the urine, with further formation of stones, development of nephrocalcinosis and finally, renal failure. The lower the renal function, the more generalized is the disease, and the deposition of oxalates accelerates when glomerular filtration rate (GFR) declines below 40–50 ml/min/1.73 m^2^. The most rapid progress to renal failure is seen in young children with infantile PH1. The OxaEurope expert group (Cochat et al.) recommends planning pre-emptive organ transplantation at chronic kidney disease (CKD) stage 3b to avoid the complications of systemic oxalosis.

Experts do not recommend either isolated renal transplantation (unless there is no other option) or isolated liver transplantation [unless in selected patients (GFR 30–59 ml/min/1.73 m^2^)] [23]. The latter option (pre-emptive isolated liver transplantation) was reported as very successful by Perera et al., however the pre-transplant GFR in children who received liver grafts was over 80 ml/min/1.73 m^2^. In any case, all transplanted patients maintained renal function for many years. Another report included three cases with median GFR of 40 (30–46) ml/min/1.73 m^2^ who received pre-emptive liver transplant successfully as well [24, 25]. The OxaEurope expert group recommends CLKT or SLKT in patients with PH1, according to patient’s condition and local facilities. Patients with GFR between 15 and 29 (CKD stage 4) may be considered as candidates for SLKT (optionally), and patients with lower GFR (< 15 ml/min/1.73 m^2^; CKD stage 5), as well as small children with infantile PH1, should receive CLKT [23].

Recent advances in developing RNA interference (RNAi)-based therapeutics, targeting glycolate oxidase and hydroxyproline dehydrogenase, aimed at treating liver-related diseases (including oxalosis), may change the policy of final treatment of PH1. The new drugs (undergoing phase III trials) are able to modify oxalate turnover to the extent that they probably will replace other therapies and limit the indications for performing CLKT/SLKT in the future [26].

There are reports on failure of isolated kidney transplantation in hyperoxaluria type 2 (PH2), associated with genetic mutations resulting in decreased activity of glyoxylate reductase/hydroxypyruvate reductase (*GRHPR*). Despite the generally milder clinical course (compared to PH1) and fact that expression of this enzyme is not limited to the liver, cases (both pediatric and adult) of renal allograft failure due to recurrence of oxaluria and presence of oxalate deposits in transplanted kidneys have been described [27, 28]. In one of these (an adult case), a further CLKT was performed with further normalization of plasma oxalate within 2 weeks after transplantation. The clinical course of these cases raises the question of qualification policy to isolated kidney vs. combined liver–kidney transplantation in PH2.

### Ciliopathies

#### Autosomal recessive polycystic kidney disease (ARPKD)

Autosomal recessive polycystic kidney disease (ARPKD) is one of the ciliopathies and is caused by mutations of the *PKHD1* gene, encoding fibrocystin. There have been more than 300 mutations described and these may cause variable phenotypes, as there is no strict genotype–phenotype correlation. The underlying pathology of renal failure is related to the presence of multiple medullary and corticomedullary cysts in enlarged kidneys. Portal fibrosis and hypertension, dilatation of biliary ducts, cholangitis, and malformations of ductal plates are common hepatic pathologies. From a clinical point of view, ARPKD can be divided into four subgroups. The first group (perinatal) presents in early infancy with severe renal disease, congestive heart failure, and pulmonary hypoplasia. The second group (neonatal) presents predominantly progressive renal failure and Caroli syndrome in the liver, whereas the two remaining groups—infantile and juvenile—present milder renal disease and more severe hepatic complications, mostly expressed as hepatic fibrosis [29, 30]. Many cases (about 70–90%) may be finally treated with isolated kidney or liver transplantation; therefore the incidence of combined/sequential transplantation in patients with ARPKD is about 10–30%. In cases of failure of both organs (developing sequentially or present in parallel)—the therapeutic options include SLKT in younger children (kidney first, then liver), whereas CLKT or SLKT (liver first, then kidney) are performed mainly in older patients (infantile and juvenile variants of ARPKD) [31–33]. There have been specific concerns reported in ARPKD patients treated with organ transplantation: one was the increased risk of ascending cholangitis in native liver after isolated renal transplantation, especially in cases with a phenotype of Caroli disease (up to 10 times higher compared to cases with hepatic fibrosis), developing as the consequence of bacterial infection, exacerbated by immunosuppression given after renal transplantation; the second issue is increased sepsis-related mortality [34–36]. Therefore, qualification for CLKT/SLKT in ARPKD should be decided carefully, on an individual basis, based on multidisciplinary discussion and dependant upon center experience. The presence of irreversible renal and liver failure with relevant complications (despite specific treatment), together with PELD score (> 10) has been suggested as the algorithm for management of children with ARPKD in terms of CLKT [37]. However, most ARPKD patients present preserved synthetic liver function, therefore the PELD scoring system may not reflect the optimal criterion for CLKT, so other factors, including therapy-resistant portal hypertension, ascending cholangitis or pruritus should also be considered [1]. An experienced group reported three (out of eight in the series) patients with ARPKD, who received pre-emptive CLKT at CKD stage 4 (eGFR about 20 ml/min/1.73 m^2^) with successful outcome, showing that the rule of qualification may be individual. Moreover, successful CLKT was associated with significant improvement of life quality and improvement of growth velocity in this series [21]. The availability of body size-matched deceased donors of kidney and liver is one of the important issues, especially in small recipients, even though large kidneys and native liver will be removed before and during final transplantation.

#### Nephronophthisis associated with liver fibrosis (Boichis syndrome)

Up to 5% of patients with nephronophthisis present hepatic fibrosis and this clinical pattern is known as Boichis syndrome. The relevant genetic background (mutation of the *TMEM67* gene) has been identified [38, 39]. The French multicenter group reported three cases of this syndrome (among overall 18 in the series of transplanted patients) who received CKLT [40]. Sequential transplantation (SLKT) was also reported in a patient with Boichis syndrome, interestingly with both organs coming from the same living-related donor. The liver transplantation was complicated with rapid progression of chronic renal failure and 4 months later the kidney was transplanted. Both grafts were functioning at 1.5-year follow-up [15].

### Methylmalonic acidemia (MMA)

Methylmalonic acidemia (MMA) is heterogenous inborn error of metabolism most typically caused by mutations in the vitamin-B12-dependent enzyme methylmalonyl- CoA – mutase (*MUT*). The defect of this enzyme leads to multiorgan failure and disability caused by chronic intoxication with methylmalonic acid and creating megamitochondria in affected organs [41]. As the overall survival of the patients has improved, renal failure is currently more frequently reported as a long-term complication [42]. Organ transplantation is, however, a therapeutic, not a curative option, as some systemic dysfunctions (such as neurologic or muscle impairment) may persist despite normal graft function. It is crucial to maintain the specific high-calorie diet for life, which is low in propiogenic amino acid precursors, as well as regular metabolic monitoring, despite transplantation, because of ongoing extrahepatorenal production of methylmalonic acid derived from skeletal muscles [42]. Some reported patients received isolated liver transplants, some isolated renal transplant, and the others underwent CLKT [43–46]. Pre-emptive isolated renal transplantation was also suggested as a form of “cell therapy”, however overall experience with this form of therapy was poor, with more complications than benefit for the patients [42]. There are reports on isolated liver and CLKT in children with MMA. Several patients undergoing CLKT were nephrectomized. Parenteral nutrition containing amino acids is an important part of post-operative management [43, 45]. Optimal transplant modality in patients with MMA is still not clear. The risks and complexity of CLKT (especially in young recipients) must be balanced with prolonged exposure to complications of the native disease if the qualification to transplantation is postponed [43].

### α1-antitrypsin deficiency (α-AT)

α1-antitrypsin deficiency (α1-AT) is a genetic disease caused by mutations in the gene *SERPINA1* and in cases of homozygous mutation children develop end-stage liver failure requiring liver transplantation [46]. Membrano-proliferative glomerulonephritis (MPGN) is one of the associated co-morbidities, which rarely may progress to renal failure [47]. The precise mechanism of this association in not fully clarified, although it has been suggested that abnormal protein released from injured hepatocytes may act as an antigen, which triggers an immunological response and leads to deposition of antitrypsin-containing deposits in glomeruli [48]. Isolated liver transplantation may cause disappearance of MPGN in native kidneys and this should be considered in qualification to isolated liver transplantation or CLKT [49]. Successful CLKT was reported in a case of end-stage renal failure in the course of MPGN in a patient with liver failure due to α-AT deficiency [48].

### Type 1a glycogen storage disease (von Gierke disease)

Type 1a glycogen storage disease (GSD1) is an autosomal recessive disorder with deficiency of glucose-6 phosphatase and related multiorgan accumulation of glycogen resulting in multiple dysfunctions, including hepatomegaly and liver failure, development of hepatocellular adenomas, and renal failure in the course of secondary glomerulosclerosis. Two successful case reports of CKLT in GSD1 have been published [50, 51].

### Atypical hemolytic uremic syndrome (aHUS)

Combined liver–kidney transplantation was used as a therapeutic option in patients with genetic aHUS at high risk of recurrence before the availability of anti-C5 monoclonal antibody (eculizumab), and the relevant recommendations concerning eligibility of patients for CLKT or isolated liver transplantation have been released by the consensus group. Successful CLKT may be curative in aHUS, however this procedure is not free from significant complications and peri-transplant overall mortality rate is about 15% [52–54]. Currently, with increasing experience in the efficacy of long-term administration of eculizumab, which prevents the recurrence of aHUS after isolated renal transplantation, CLKT was abandoned as first-line treatment option in most relevant cases [55, 56]. The availability of these expensive drugs is increasing and in many countries the drug company-driven program of repeated i.v. administration at home is helpful in cases of necessary long-term prophylaxis. In rare cases of suboptimal response to eculizumab, sequential liver after kidney transplantation may be a curative option, as recently reported by an Italian group [57]. The definitive recommendation on considering (or not) CLKT as a curative option in genetic aHUS is not yet available and longer follow-up of eculizumab-driven cases with isolated renal transplantation should bring more evidence, which will make this decision more reasonable.

## Renal failure after liver transplantation

There are two distinct subgroups of patients who develop renal failure after liver transplantation. One includes patients who received liver transplant having developed CKD at stage 4/5 or developing chronic post-acute renal failure in the course of hepatorenal syndrome (HRS). The second group includes patients with normal renal function at the moment of liver transplantation, but who develop chronic kidney injury several years thereafter, mainly due to nephrotoxicity of calcineurin inhibitors (CNI). There are no specific pediatric recommendations regarding this, however there is ongoing discussion on optimal therapy in the first setting, conducted among transplant physicians and surgeons treating adult patients. The consensus guidelines divide (adult) candidates for CLKT into two renal categories: (1) persistent acute kidney injury (AKI) ≥4 weeks, or eGFR ≤35 ml/min (by MDRD-6 formula) or eGFR ≤25 ml/min (by iothalamate clearance), and (2) CKD for 3 months before qualification, defined as eGFR ≤40 ml/min (by MDRD-6 formula) or eGFR ≤30 ml/min (by iothalamate clearance) or proteinuria ≥2 g/day or chronic pathology in renal biopsy (> 30% global glomerulosclerosis or > 30% interstitial fibrosis) or metabolic disease involving kidneys [58]. These criteria reflect experience showing better outcomes in CLKT vs. isolated liver transplantation in adult cirrhotic patients with pre-existing renal failure [59]. More recent data are more careful in terms of qualification rules, and show that the real benefit in patient survival after CLKT is limited to those cases who required dialysis before transplantation [60]. In children with liver failure and after liver transplantation, the modified Schwartz formula may overestimate renal function, therefore cystatin C-based calculation of eGFR is indicated for more accuracy [61, 62]. Children with end-stage renal disease developing as a consequence of hepatorenal syndrome will be apparent candidates for SLKT. The other subgroup of indications includes patients with liver failure, who develop CKD, then end-stage renal disease after many years after liver transplantation and require renal transplantation (SLKT). The incidence of GFR decrease < 90 ml/min/1.73 m^2^ at 5–10 years after liver transplantation is about 30% [62], however the development of end-stage renal disease is reported as below 10% in children after long-term follow-up [63]. There are preventive measures undertaken to avoid renal transplantation for this reason. The first includes careful detailed surveillance of renal function and blood pressure in pediatric liver transplant recipients [64] and the second is aimed to minimize exposure to CNI [61]. There have been several randomized controlled trials aimed at reducing the risk of CKD in adult liver transplant recipients, based on conversion from CNI to mTOR inhibitors. A recent meta-analysis showed that patients who converted to mTORi had significantly better renal function at 1 year after randomization compared with patients remaining on CNI (mean difference, 7.48 ml/min/1.73 m^2^). However, conversion to mTORi was associated with a higher risk of acute rejection (RR, 1.76) and study discontinuation due to adverse events (RR, 2.17) up to 1 year after randomization [65]. A similar approach was reported recently in children, where treatment with everolimus and low-dose CNI revealed over-immunosuppression associated with use of this particular protocol and recruitment was stopped prematurely due to high rates of PTLD, treatment-related serious infections leading to hospitalization and premature study drug discontinuation, despite significant benefits in terms of improvement of GFR [66]. Clinical factors used for qualification of patients CLKT/SLKT are presented in Table 2.

**Table 2:** Factors used for qualification to CLKT/SLKT

Indication	Factors	Citation
Hyperoxaluria type I (PH1)	High oxalate burden, not responding to pharmacological treatment CKD stage 4: eGFR < 29 ml/min/1.73 m^2^ (optional; SLKT) CKD stage 5: eGFR < 15 ml/min/1.73 m^2^ (direct; CLKT)	[23–25]
Autosomal recessive polycystic kidney disease (ARPKD)	PELD score > 10 (age, albumin, bilirubin, INR, growth failure) and therapy-resistant portal hypertension/ascending cholangitis/pruritus Complications of portal hypertension Complications of biliary tract (cholangitis) Severe cirrhosis in liver biopsy CKD stage 5: eGFR < 15 ml/min/1.73 m^2^/dialysis (CLKT) Availability of size-matched deceased donor of liver and kidney	[31–37]
CKD after liver transplantation	Hepatorenal syndrome (optional for SLKT), if dialysis after liver transplantation needed for > 6 weeks (renal biopsy needed for final decision) CKD stage 4: eGFR < 30 ml/min/1.73 m^2^ or CKD stage 5: eGFR < 15 ml/min/1.73 m^2^ Severe chronic damage in renal biopsy (tubulointerstitial fibrosis > 35%; glomerulosclerosis > 35%) (SLKT) * *data from adult setting	[58, 60–63]

*ARPKD* autosomal recessive polycystic kidney disease, *PELD* pediatric end-stage liver disease (score), *INR* international normalized ratio, *CKD* chronic kidney disease, *CLKT* combined liver–kidney transplantation, *SLKT* sequential liver–kidney transplantation; *eGFR* estimated glomerular filtration rate, *PH1* primary hyperoxaluria type I

## Outcomes

There are variable data on long-term outcomes of CLKT, depending on the series of patients and specific indications (when compared to outcomes of isolated organ transplantation). The major difference is also related to the primary parameter of outcome: - incidence of acute rejection, or - patient and graft survival. Overall, the incidence of acute rejection in CKLT is lower compared to isolated liver or kidney transplantation (reports from UNOS and CTS databases) [7, 14], which supports the idea of an immunoprotective effect of the transplanted liver to the kidney coming from the same donor. The ESPN/ERA-EDTA Registry data on outcomes of CLKT in children with ARPKD shows that 5-year patient survival after kidney transplantation is 97.4%, in contrast to 87.0% after combined liver–kidney transplantation. The age- and sex-adjusted risk for death after combined liver–kidney transplantation was 6.7-fold greater than after kidney transplantation (*P* = 0.005) [1]. These data also show that 5-year death-censored kidney transplant survival following combined liver–kidney and kidney transplantation was similar (92.1 vs. 85.9%; *P* = 0.4), which is in contrast to the opinion of a protective effect of the liver on the renal graft. Overall, the early post-transplant period in CLKT is associated with high risk for patients (mainly small children), while long-term graft survival is not inferior compared to isolated transplantations. Data from the Scientific Registry of Transplant Recipients (SRTR), including 152 primary cases of CLKT (1987–2011), shows that patient survival was 86.8, 82.1, and 78.9%, liver graft survival was 81.9, 76.5, and 72.6% and renal graft survival 83.4, 76.5, and 66.8% at 1, 2, and 10 years, respectively. Similar survival rates for isolated liver transplantations were 86.7, 81.2, and 77.4%, and for isolated kidney transplantations 98.2, 95.4, and 90%, respectively. In patients undergoing CLKT, PH1 as the underlying disease (the most common indication; 37% of all CLKT cases) was associated with reduced patient, liver, and kidney graft survival (*p* = 0.01; *p* = 0.01; *p* = 0.01; respectively). The study also showed that during one decade, graft outcome after CLKT significantly improved (*p* = 0.04 for liver and *p* = 0.02 for kidney transplantation), however this did not translate into better patient survival (*p* = 0.2). The study demonstrated the center effect on overall patient survival, with inferior outcomes in children receiving CLKT in centers with minor experience (defined as 1–2 CLKTs performed) [22]. Overall, 100% 1- and 5-year (death-censored) liver and kidney graft survival, and 94% 5-year patient survival in children with ARPKD treated with CLKT was reported by our center, which is an active national pediatric transplant center [13]. The UNOS registry data on patient and graft survival in children with SLKT (liver transplant recipients subsequently receiving renal graft) showed that overall patient survival was inferior (90% at 5 years and 82% at 10 years; *p* = 0.002), however (death-censored) graft survival was similar compared to isolated kidney recipients (82% at 5 years and 67% at 10 years; *p* = 0.65) [67]. These data suggest the overall high risk of multiorgan transplantation, also in the setting of sequential procedures, compared to isolated transplantation.

## Key summary points

• Indications for combined or sequential liver–kidney transplantation (CLKT/SLKT) include parallel or sequential failure of both organs, the need to replace the liver as the source of a specific missing enzyme in a patient with renal failure, and the need for renal transplantation in the recipient of liver graft due to renal failure.

• The selection of optimal approach (CLKT vs. SLKT) is individual depending on specific clinical indications, current status of both organs, general condition of the recipient, and experience of the center.

• In CLKT, the liver exerts a protective immunological effect upon the kidney, which translates to low acute rejection rates and good long-term graft survival, however this effect seems to be limited to non-sensitized patients.

• In SLKT, there is no protective effect, as in most cases liver and kidney come from different donors, however the HLA matching between sequential organs (liver and kidney) is important for future outcome.

• Overall outcome of CLKT/SLKT is good in terms of long-term graft survival, however it is not superior to an isolated liver or kidney transplantation setting in terms of patient survival, which is related to the complexity of transplantation procedures, especially in young children and the high risk during the early perioperative period.

## Questions (answers are provided following the reference list)

1. Qualification for organ transplantation in children with primary type 1 hyperoxaluria (PH1) is based on:

- Basic rule: ‘kidney first’ in all cases
- Basic rule ‘liver first’ in all cases
- Evaluation of GFR and adjusting strategy to the stage of CKD
- Degree of liver failure

2. Transplanted liver protects renal graft against acute rejection:

- Always
- When both grafts come from the same donor and the recipient is not hyperimmunized
- When the liver comes from living-related donor and the kidney from a deceased donor
- If the time interval between the sequential transplantations exceeds 1 year

3. During CLKT:

- Liver is transplanted first and then the kidney
- Biliary anastomosis is performed before kidney reperfusion
- Kidney reperfusion should precede liver reperfusion
- Optimal cold ischemia time for both organs should be not longer than 16 h

4. When patients with ARPKD receive an isolated renal transplant:

- Liver function improves
- There is no effect of this procedure on native liver
- Liver volume decreases due to better fluid turnover
- There is a risk of recurrent cholangitis in the native liver, exacerbated by post-transplant immunosuppression

5. CLKT in atypical hemolytic uremic syndrome (aHUS):

- Is a single effective curative procedure
- Is still widely performed, as monoclonal anti-C5 is not effective in most cases of isolated kidney transplantations
- Is indicated only when a living-related donor is available
- Is not currently regarded as first-line option, as repeated administration of monoclonal anti-C5 is effective in isolated kidney transplantation for prevention against recurrence of aHUS

6. Imediate post-CLKT extracorporeal removal of oxalate in patients with primary type 1 hyperoxaluria (PH1):

- Is unnecessary, as well functioning liver transplanted easily eliminates whole pool of oxalate
- Is indicated in cases of (renal) delayed graft function
- Is contraindicated in patients plasma oxalate concentration > 100 μmol/l and good urine output
- Should never be done due to high risk of bleeding

## Abbreviations

AXGT: Alanine-glyooxylate aminotransferase
AKI: Acute renal injury
aHUS: Atypical hemolytic uremic syndrome
anti-IL2R ab.: Antibody against receptor for interleukin 2
ARPKD: Autosomal recessive polycystic kidney disease
AZA: Azathioprine
CKD: Chronic kidney disease
CLKT: Combined liver–kidney transplantation
CTS: Collaboration Transplant Study
CNI: Calcineurin inhibitors
CsA: Cyclosporine
CVVHDF: Continuous veno-venous hemodiafiltration
CVP: Central venous pressure
eGFR: Estimated glomerular filtration rate
GRHPR: Glyoxylate reductase/hydroxypyruvate reductase
GSD-1: Glycogen storage disease type 1a
HLA: Human leukocyte antigens
HRS: Hepatorenal syndrome
INR: International normalized ratio
IVC: Inferior vena cava
MDRD (equation): Modification of diet in renal disease
MMA: Methylmalonic acidemia
MMF: Mycophenolate mofetil
MPGN: Membrano-proliferative glomerulonephritis
NK: Natural killer cells
PELD: Pediatric end-stage liver disease (score)
PH1/PH2: Primary hyperoxaluria type 1 and type 2
Pred: Prednisolone
RIFLE (scale): Risk/injury/failure/ loss/ end-stage renal failure
RNAi: Ribonucleic acid interference
SIR: Sirolimus
SLKT: Sequential liver–kidney transplantation
SVC: Superior vena cava
TAC: Tacrolimus
UNOS: United Network of Organ Sharing
